# Towards a Mobile-Based Platform for Traceability Control and Hazard Analysis in the Context of Parenteral Nutrition: Description of a Framework and a Prototype App

**DOI:** 10.2196/resprot.4907

**Published:** 2016-06-07

**Authors:** Víctor M Alonso Rorís, Luis M Álvarez Sabucedo, Carmina Wanden-Berghe, Juan M Santos Gago, Javier Sanz-Valero

**Affiliations:** ^1^ E E Telecomunicación University of Vigo Vigo Spain; ^2^ Foundation for the Promotion of Health and Biomedical Research from the Valencian Community (FISABIO) University General Hospital of Alicante Alicante Spain; ^3^ Department of Public Health & History of Science University Miguel Hernandez Alicante Spain

**Keywords:** parenteral nutrition, quality control, process assessment, information management

## Abstract

**Background:**

The parenteral nutrient (PN) mixtures may pose great risks of physical, microbiological, and chemical contamination during their preparation, storage, distribution, and administration. These potential hazards must be controlled under high levels of excellence to prevent any serious complications for the patients. As a result, management control and traceability of any of these medications is of utmost relevance for the patient care, along with ensuring treatment continuity and adherence.

**Objective:**

The aim of this study is to develop a mobile-based platform to support the control procedures and traceability services in the domain of parenteral nutrient (PN) mixtures in an efficient and nonintrusive manner.

**Methods:**

A comprehensive approach combining techniques of software engineering and knowledge engineering was used for the characterization of the framework. Local try-outs for evaluation were performed in a number of application areas, carrying out a test/retest monitoring to detect possible errors or conflicts in different contexts and control processes throughout the entire cycle of PN. From these data, the absolute and relative frequencies (percentages) were calculated.

**Results:**

A mobile application for the Android operating system was developed. This application allows reading different types of tags and interacts with the local server according to a proposed model. Also, through an internal caching mechanism, the availability of the system is preserved even in the event of problems with the network connection. A set of 1040 test traces were generated for the assessment of the system under various environments tested. Among those, 102 traces (9.81%) involved conflictive situations that were properly taken care of in this paper by suggesting solutions to overcome them.

**Conclusions:**

A mobile oriented system was generated and tested in order to allow enhanced control and quality management of PN mixtures that is easy to integrate into the daily praxis of health care processes.

## Introduction

Mixtures of parenteral nutrition (PN) enable intravenous delivery of essential nutrients to patients who cannot be fully fed orally. The PN mixture may contain more than 50 components with a high potential for physicochemical interaction among its ingredients, the bag, oxygen, temperature, and light. These interactions are potentially iatrogenic and, in some cases, may even compromise the patient’s life [1,2]. Therefore, PN is considered a high-alert medication that must be controlled throughout its life cycle [3].

In such contexts, in order to minimize errors and problems in the procedures involved, clinical practice guidelines and recommendations for action are generated [4]. A properly defined protocol ensures a high degree of theoretical viability, quality, and safety of the intended process. Thus, it is possible to adapt the context to strict quality requirements of the health care environment and compliance, in the elaboration and control of PN, with current regulations [5].

The criterion that determines whether those goals are achieved is adherence to the protocol of the different operations performed by the agents involved. Thus, in this type of high-risk context, to ensure proper execution of the processes, it is mandatory to check that the protocols are being applied and the results are the expected ones. In line with this, certain time points and places within processes need to be monitored. These elements are known as control points (CP) and allow for verification of the defined requirements [6]. Additionally, depending on the probability of the event, and especially the severity of the potential damage, those CP that require special supervision, which are referred to as critical control points (CCP), should be identified [7].

At the CP, and especially the CCP, data records from the applied monitoring system are generated, which are known as traces [8]. Gathered traces allow explicit statement to be made about the states or interactions, or both, between the different elements involved in the various processes that are being carried out. A thorough and complete log of traces makes full awareness of the history, usage, or location of an entity possible. This ability is known as traceability [9].

In the context of PN, traceability makes following the movement of a mixture through all the stages of its life cycle possible. Specifically, traceability is aimed at determining with certainty which vendors and products are part of a PN mixture’s composition (traceability back), tracking the PN in production time (internal traceability), and tracing the mixture once it is produced and distributed (traceability forward) [10]. Thus, an efficient system of traceability should be able to react quickly and appropriately to any quality risks identified or to hazards related to the safety of medicines.

Various technological proposals are focused on traceability, particularly in the logistics area [11]. But, unfortunately, this type of deployment is based on automatic recording from the temporary location of the product and cannot cover all immediate demands, requirements, and needs of comprehensive management control in a context such as that of the PN. Nevertheless, mobile technologies are providing new solutions, based on comprehensive patient-focused care models, which make them very attractive for clinical care applications [12,13].

Having noted the benefits of a management and traceability platform for PN using mobile technologies, we proposed the design and implementation of a holistic service architectural framework to fill the gaps in traceability and to provide flexible and effective telematic mechanisms for monitoring PN in a health care setting. We describe a technology platform to support control procedures and traceability of PN mixtures in an efficient and nonintrusive way.

## Methods

### Models

We used a comprehensive approach combining software engineering and knowledge engineering techniques to characterize our framework. In particular, we generated three initial models to fully define the system from different perspectives. Together they provide a complete description of the solution. We tested these models [14], which are a business model (high-level description of conditions, agents, and general behavior of the platform); a semantic model (formalization of the knowledge within the system using technologies that facilitate automatic processing and interpretation of information); and a reference architecture (the definition of a framework for the development of a software solution).

#### Business Model

The business model is intended to identify the basic features of the technological platform. This characterization offers a vision, not necessarily formal, of how the whole system works in this context.

To conduct this modelling, in addition to consulting the opinion of experts, we reviewed the available literature (science, technology, legal, etc) and generated different usage scenarios for analysis and validation.

By using this model, we were able to identify, manage, and freely modify on run time the data attached to CP, CCP, and the monitoring parameters associated with the entire life cycle of a PN mixture. We also labelled the elements involved in the context (pumps, filters, etc) with matrix barcodes (quick response, or QR, and data matrix codes) and near field communication (NFC) tags to identify them uniquely.

#### Semantic Model

The semantic model formalized the proposed traceability mechanisms, which facilitated the analysis and automatic processing of the history of each PN mixture. In this sense, it was essential to establish a formal scheme that would describe the information in a way that would enable the efficient application of reasoning or complex queries.

This modelling allowed formal conceptualization of the universe of applications and of relationships, which would be easily readable and interpretable by machines. Having semantic support in the model enabled the application of advanced information technologies and added-value solutions and tools, such as inference engines that perform logical reasoning (or make inferences). Such reasoning enabled us to extract new facts and knowledge and to answer specific and complex queries.

Following this line of reasoning, we defined an open and generic data model focused on the definition of common concepts (with their properties) for related scopes of the domain of interest: users, institutions, CP, CCP, services, and parameters to monitor, for example traces and context variables. This semantic model provided us with an abstract representation of the whole application domain. To this end, we generated specific internal vocabularies and, as much as possible, reused vocabularies that are widely accepted in the field of semantics (eg, friend of a friend, resource description framework schema).

Uniform resource identifiers (URIs) [15] univocally identify semantic concepts and entities. This usage is derived from the HTTP paradigm and makes it possible to reference resources. This feature allowed us to attach the URI of each element in the semantic model to the information on the label on each actual item. Thus, by reading a label, regardless of the agent involved, it was possible to directly access a representation of the information stored in the system for the specific entity.

#### Reference Architecture

The next step in the comprehensive modelling of the system was to define a reference architecture that would provide a guide or framework for the development and final implementation of the software platform. In general, we used a client-server architecture. Thus, the business logic was hosted on servers accessible over the network and, as a result, the complexity of the agents was simplified.

In this methodology, the reference architecture is described from different perspectives that compose a comprehensive view of the platform. The aim is to be able to speak to the characteristics and peculiarities defined in the business model and the semantic modelling.

##### Client-Server Interaction

**Figure 1:**
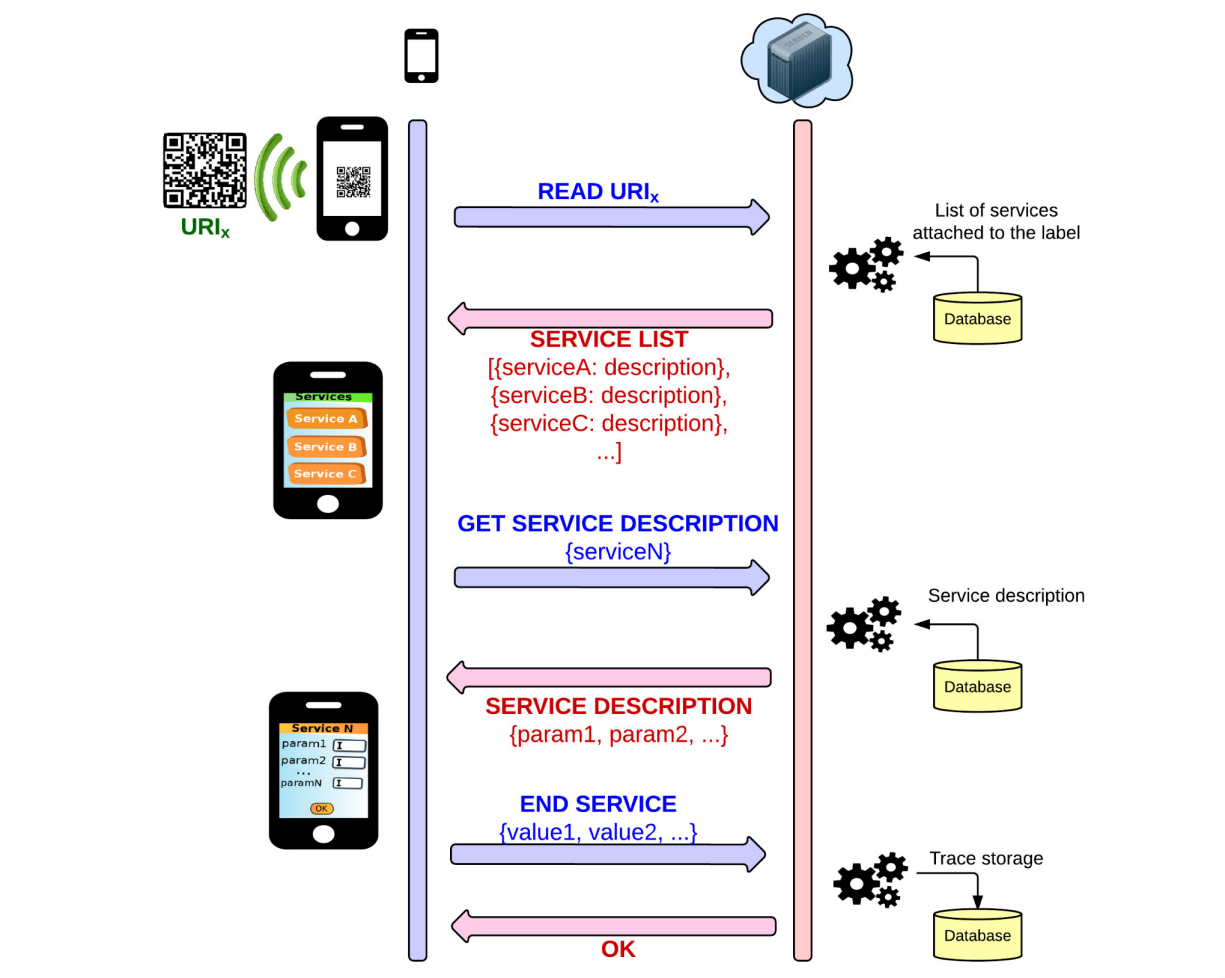
Model representation of a regular interaction between client (software) and server.

To carry out a successful interaction with the frame of the proposal, the client (the software that runs on the end user device) and server must interact according to the established model. In particular, this interaction must be compliant with the model presented in Figure 1. In this example, after reading the label on a PN bag, the client launches a request to the server. The server then responds with a list of services associated with the element depending on the requesting user and other related variables of context.

Afterward, the client must select the actual service to invoke using an on-screen interface. On selection, the server sends back a full description of the service under consideration. Using this description, the client software generates a form in the display with the parameters required by the service and that the user must provide. Finally, the values entered into the form are sent to the server according to the software interface and become a trace in the system.

##### Reference Model

During the life cycle of a PN mixture, management and control of the traces may concern different institutions or companies (eg, pharmaceutical industry, hospitals, transport companies). Given the sensitive nature of the information processed, these organizations are compelled to store the information generated in its ecosystem under rigorous security measures to ensure compliance with legal and ethical regulations.

To achieve the required security levels, each organization may deploy its system locally according to its own standards of security. This decentralized scenario is achieved through the use of specialized agents (running mobile apps for the final clients) that have the appropriate logic to establish direct connections with servers in their organizations.

**Figure 2:**
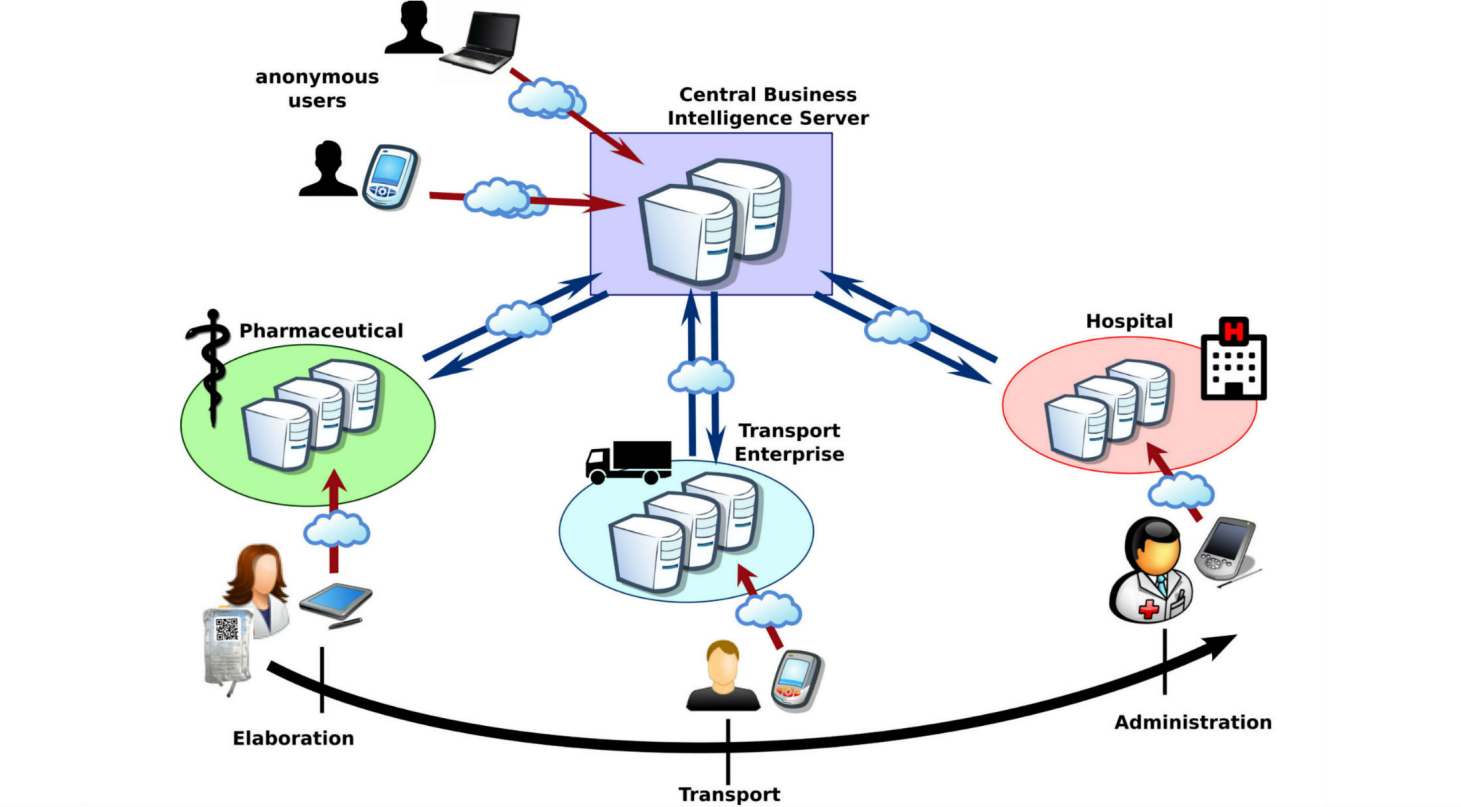
Reference structure for the different components and communication models.

By means of this modelling, different servers may record different traces throughout the life cycle of the product (see Figure 2). Consequently, if an unregistered user accesses the system, the central server is responsible for redirecting the request to the proper agent that possesses the requested information.

The central server acts, from the point of the client agent, as the single access point and coordinator for the existing local system. In this way, the fragmented system appears seamless to the user.

#### Modular Architecture

**Figure 3:**
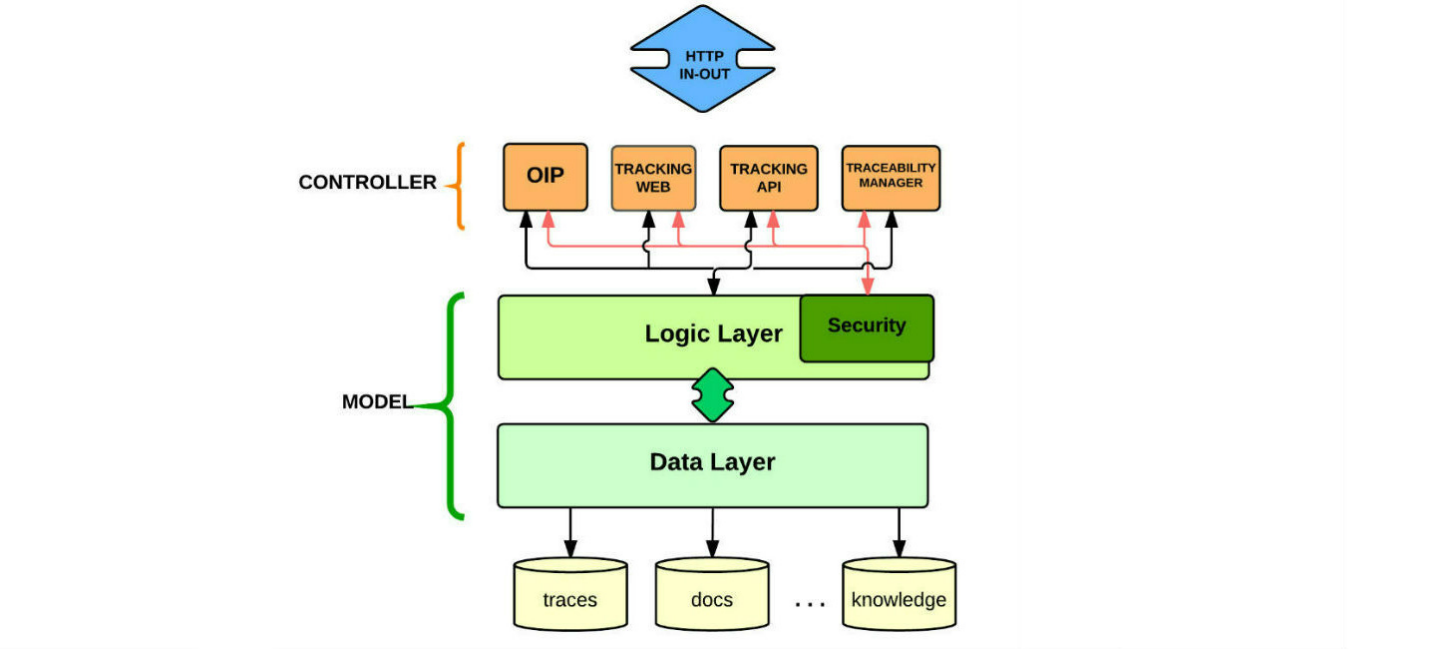
General architecture including server modules.

The architecture model for the server was designed following a modular approach. Thus, it was possible to divide the overall complexity of the system, and the logic layer was distributed according to the nature of each component. Figure 3 shows the high-level architecture of a server and different interfaces and management layers. We describe the main modules below.

##### Open Information Provider

This provides a universal access point for public information about semantic records. In particular, it responds to HTTP requests on the URI that uniquely identifies the elements within the domain of the application.

##### Tracking Application Programming Interface

This provides the functionality that is available for interaction with the specialized customer software. These features include services for discovering control services, accessing information about a concrete item, downloading service descriptions (eg, describing how to monitor variables), invoking a specific service for registration of the relevant traces, and recovering useful material for human users (videos, manuals, etc).

##### Tracking Web

This provides similar functionalities to those offered by the tracking application programming interface using just the support of a Web browser.

##### Traceability Manager

This provides a Web interface designed to support 1) functionalities related to traceability, such as checking historical traces according to user preferences, date, location, and related services, 2) an advanced search engine supporting advanced queries, 3) facilitating the definition of logical inference patterns and automatic analysis of procedures, problems, alerts, etc, 4) statistical analysis of existing traces to enable high-value services, and 5) automatic verification of adherence to protocols according to existing traces.

### Evaluation of the Platform

We developed simulation testbeds to test the validity of the prototype we developed. A test-retest control detected possible errors or conflicts in different contexts and control processes throughout the entire cycle of the PN mixture. From these data, we calculated the absolute and relative frequencies (percentages). We controlled information quality by double entering data; we corrected inconsistencies by consulting the original data.

## Results

### Prototype

We generated a mobile app for the Android operating system according to our presented models. This app reads different types of labels and tags, and interacts with the local server under the proposed schema. Also, the above-mentioned app ensures operability of the system, even in case of network disconnection, by means of an internal caching mechanism.

During the prototype implementation process, the following elements were provided: the central server, to redirect requests to the proper local server responsible; the local servers, to provide the necessary functionality for control and traceability; and the client app that supports efficient mechanisms for the discovery of CP and CCP.

**Figure 4:**
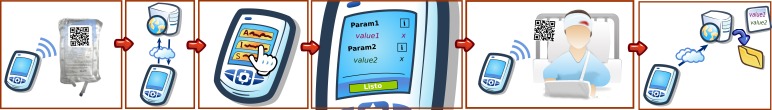
Outlining the interaction with the platform through the QR code attached to a bag of PN mixture.

Within the frame of the proposed model (see Figure 4), on reading the QR tag associated with a nutrition bag, the client software retrieves the list of invokeable services (offered by the system) and displays them to the user. To select the desired service, the user accesses a form on the device screen to submit the variables to be monitored. Finally, the information is sent to the server, which processes the new information and records it as a trace.

In this way, after reading a label attached to a PN bag using a mobile device with Internet access, the user can discover the control operations related to the identified element, at run time. That is, the platform dynamically retrieves control operations that can be invoked by the user according to the role assigned in the system (nurse, pharmacist, doctor, etc), the element under consideration, and other context variables such as time and location. All collected data are stored on a server that acts as a repository of information. And, using data mining techniques, the server can reuse these data and generate new knowledge by applying inference and discovery processes.

**Figure 5:**
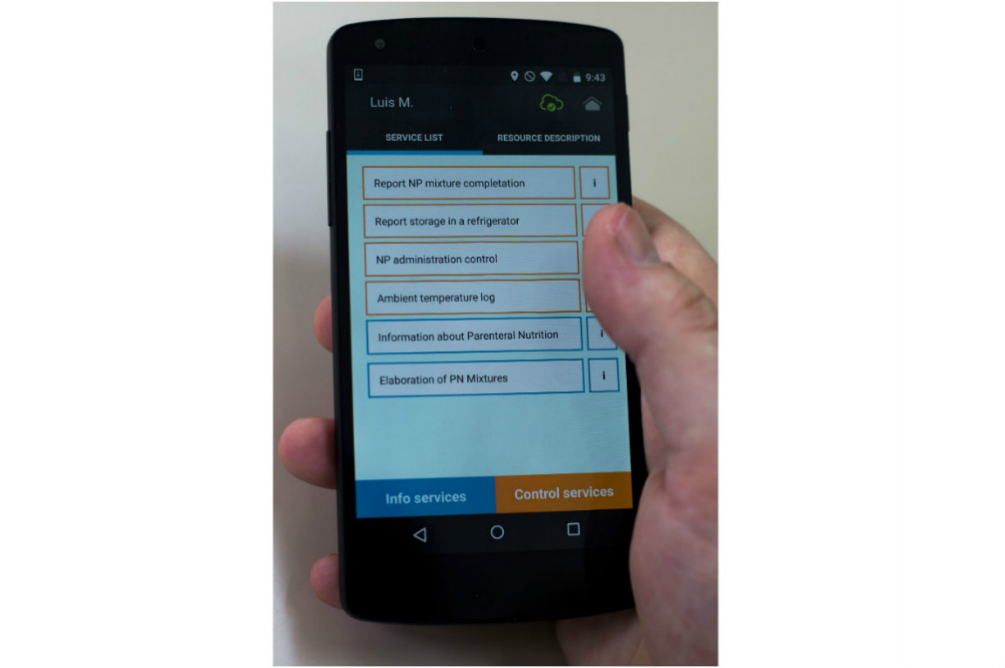
Screenshot of the mobile application showing the interface for selecting associated services to a concrete label.

**Figure 6:**
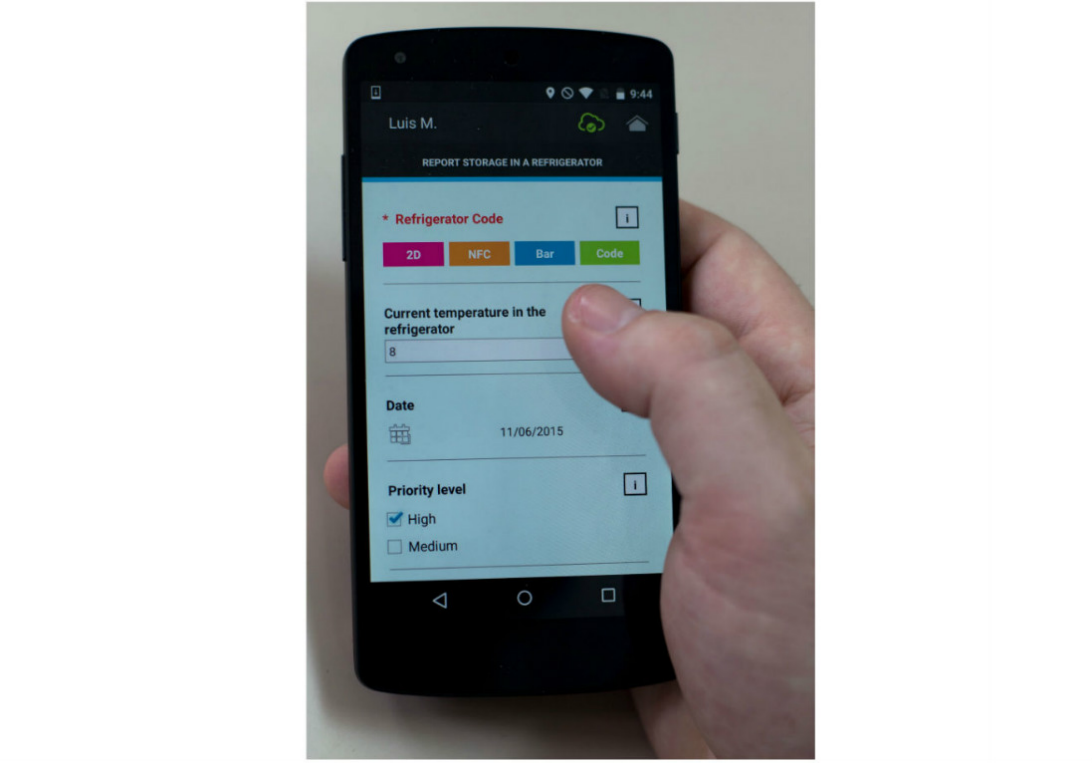
Screenshot of the mobile application showing the form for invoking a control service.

Use of the software is very intuitive and user centric, as the user interfaces are generated dynamically and ad hoc according to the descriptions provided by the server. Figure 5, for example, shows the list of services that the user can access after reading a label as the software agent generates it. In the same way, Figure 6 illustrates how the dynamically generated form is displayed to invoke service control.

**Figure 7:**
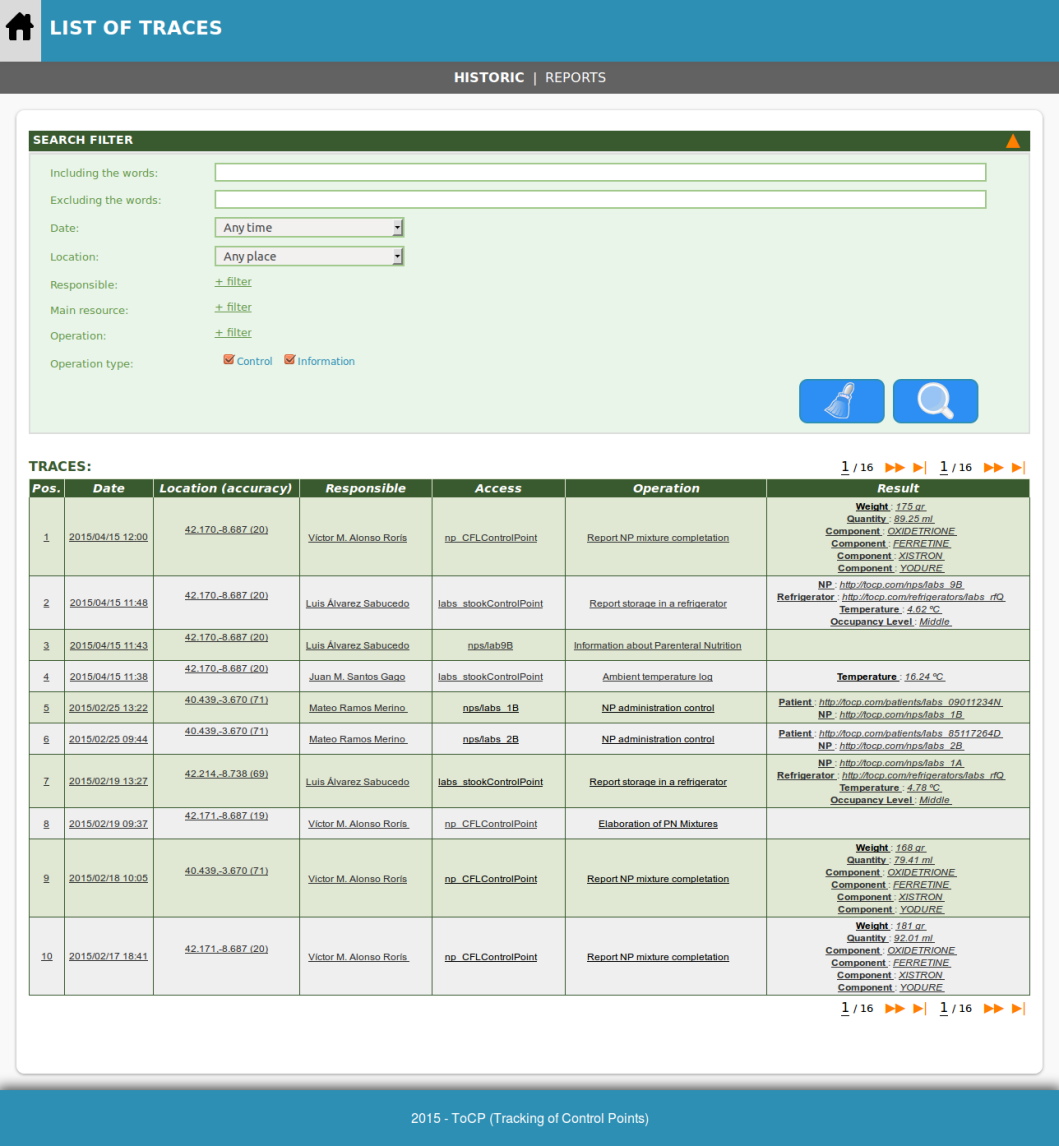
Screenshot regarding traceability functionality showing the trace log according to the search filter options.

Summing up, the developed prototype server covers the functionality required for the proper functioning of the overall process control and traceability support. Consequently, we have implemented mechanisms for logical reasoning that allow for the application of advanced processing methods on traces generated in this framework. Figure 7 is a screenshot showing the list of historical traces available in the system. In addition, this log of traces can be customized through various search filters (eg, prescription, type of nutrition, responsible for delivery, composition).

During the evaluation process, we collected 1040 test traces in different testbed scenarios. Table 1 lists the issues we identified, along with their proposed solutions.

To achieve the desired results and functionalities from the point of view of the final users, we identified several functional and nonfunctional requirements.

**Table 1:** Frequency (f) and percentage of errors (undesired situations) identified in the assessment of the management and traceability platform and their suggested corrections.

Undesired situation	f_0_	%	Solution
The QR^a^ tag was stained and not readable.	9	0.87	Laminate labels.
QR tag was attached to a surface with a large curvature and was not readable (the camera was not able to capture a defined image of the whole QR code).	11	1.06	Reduce the size of the label to minimize the curvature radius or change its location to a nearby flat surface.
In poorly lit places, the camera was not able to read the QR tag.	5	0.48	While reading, the app triggers a flash to illuminate the corresponding surface.
The NFC^b^ tag could not be read because it was attached to a metallic surface.	9	0.87	Replace basic NFC tags with special NFC tags designed to be used on metallic surfaces or place them on another spot.
The NFC tag was located behind a plastic sign on the door and could not be read.	14	1.35	Remove any surface between the mobile and NFC tag.
User was unable to log on to the app when offline the first time the app was used on that mobile device (no preexisting cache contents).	6	0.58	The system requests the user to log in, at least the first time, in an online context.
The user, trying to read the tag, noted that the battery was drained	2	0.19	User training (no solution in the app).
The user typed a decimal value by entering a comma instead of a period. As a result, data were misinterpreted.	21	2.02	Unblock use of the comma button in the app.
The system noted that it was impossible to invoke the service when the form was including incorrect values. No hints on the wrong values were provided.	6	0.58	Update the app to display a warning message when wrong data are detected. Also, present information about accurate expected values.
In the input form, when trying to send multiple documents (eg photographs) with the same name, the server could only recover the last one.	4	0.38	Implement a mechanism in the app to avoid file name conflicts.
Leaving the input form by mistake was possible, resulting in losing all data entered so far.	1	0.10	Modify the app so that trying to leave the input form prompts a message that the entered data would be lost and that requests explicit confirmation to leave the form.
The implementation required activating the GPS^c^ service to inform the server of the location of the operator in each of the traces generated. This circumstance caused high battery consumption.	14	1.35	Replace the initial mechanism to recover coordinates based on continuous queries with an intelligent mechanism that checks the GPS service according to the user’s mobility over time.
Total	102	9.81	

^a^QR: quick response.

^b^NFC: near field communication.

^c^GPS: global positioning system.

### Requirements

#### Simple and Unobtrusive Use

Use of the platform should fit into the existing standard operating procedures, which should not intrude on or misuse the platform.

#### Availability Throughout the Entire Life Cycle

The platform must include a highly adaptable and configurable framework. Thus, it would be possible to support a large range of organizations and to enable them to establish and manage their own procedures and services for the whole life cycle of the products.

#### Support for Different Types of Labels

The labels we used allowed for unique identification of the elements in the domain. Due to their popularity and low cost, we used 2-dimensional labels (QR and data matrix code) and NFC.

#### Mobile Devices

Smart mobile devices (mobile phones and tablets) offer the capacity and portability required for client use. Moreover, they are simple and intuitive to use and are highly available among potential users.

#### Different Types of Services

Services that could be generated and supported within the platform were of two types: control and information. Control services allow monitoring of one or more variables, while information services provide access to suitable pieces of information relevant to the context.

#### Support for Different Roles

The platform provides customization mechanisms. Thus, it was possible to filter the services available according to the role of the current user.

#### Security

Given the sensitive nature of the information exchanged, it is essential that the platform offer mechanisms to ensure the confidentiality of the exchanged information.

#### Offline Operability

Due to the nature of the problem, it is of the utmost relevance that no trace be lost at any point of the processes being monitored. Therefore, we developed software capabilities to make use of the platform possible even without a network connection.

## Discussion

### Principal Findings

We outlined the provision of a holistic computerized control and verification system, for the entire process of production and distribution of a PN mixture. Our solution meets the recommendation of the consensus on the preparation of parenteral nutrient mixtures of the Spanish Society of Hospital Pharmacy [1] and is in line with clinical guidelines on guarantees and rational use of PN [16].

Nevertheless, the key point to using labels as described to uniquely identify the items under consideration in any stage is related to the platform’s application in the analysis and monitoring of hazards (by means of CCP) within a system of hazard analysis and critical control points (HACCP) implemented in the whole process of preparation and administration of PN mixtures. Intravenous therapy management is a critical process in clinical patient safety involving traceability, accountability, and security. Thus, the use of HACCP-based systems ensures excellence in its control [17]. This tag, attached to the PN bag, can be very useful in ensuring links with the prescription, reporting about its proper use according to established standards, and providing data such as elaboration, issue or expiration date, dosage, and possible side effects [18]. Regarding home care, these labels integrated into PN containers facilitate the exchange of information between caregivers and the medical team, and in emergency care this platform contributes to immediate patient identification and comprehensive knowledge of medical history. This model provides, at the same time, security features in clinical data transmission [19,20].

In addition, the software developed for the Android operating system supports an internal cache mechanism that makes regular operation of the system possible even if the network connection is not available [21]. Among its capabilities, the central server acts as a repository of publicly accessible information that enables the reuse of existing knowledge in an open and free manner. To make this feature possible, the central server periodically retrieves the public records of each of the deployed servers. Then, this information is automatically enriched with external information gathered over the Web using semantic enrichment mechanisms that we have already deployed successfully in previous work [22].

Besides features related to control management and traceability, the prototype system we developed has the following advantages: improved management of patient care and quality of care, empowerment of the actors involved, and ensured continuity of the action planned. The association of these characteristics with reducing the rate of patients with missing data and better monitoring of adherence to treatments and of iatrogenic risks has been previously proven [23]. Regarding adherence to PN treatments, it has been found that only intensive care units met established goals, with adherence being especially problematic in monitoring patients admitted to home care units [24]. This problem can be addressed by using the platform we have developed.

The evaluation tests showed a low rate for the unfavorable situations we observed. Also, we were able to solve most of them fully except for those related to lack of user training. Nevertheless, this was an expected issue. We were well aware that training and user feedback should always be present in the deployment of mobile technologies [25,26].

In addition to the good evaluation results, an advantage that must be considered is the positive adoption of mobile apps due to their low cost and simplicity of use. Previous studies have shown that these apps are feasible for real life, and are productive for clinical application [27].

### Limitations

Among possible limitations of this study, it must be mentioned that, although we conducted the evaluation in a testbed designed in a realistic way from the point of view of prescription logistics, preparation, and delivery, the overall evaluation would benefit from an actual usage scenario in a heavy load condition to ensure optimal application [28].

### Conclusions

The prototype platform is fully operational and ready to be tested in a real context. Consequently, we have generated and tested a system based on mobile technology that allows better monitoring and management of the quality of PN mixtures and that is easy to incorporate into the daily praxis of health care processes.

## Abbreviations

CP: control points
CPP: critical control points
HACCP: hazard analysis and critical control points
NFC: near field communication
PN: parenteral nutrition
QR: quick response
URI: uniform resource identifier
